# Age-Appropriate Feeding Practices in Cambodia and the Possible Influence on the Growth of the Children: A Longitudinal Study

**DOI:** 10.3390/nu12010012

**Published:** 2019-12-19

**Authors:** Gabriela Hondru, Arnaud Laillou, Frank T. Wieringa, Etienne Poirot, Jacques Berger, Dirk L. Christensen, Nanna Roos

**Affiliations:** 1 Section of Global Health, Department of Public Health, University of Copenhagen, 1353 Copenhagen, Denmark; dirklc@sund.ku.dk; 2 United Nations Children’s Fund (UNICEF), Integrated Early Childhood Development, Exchange Square, 5th Floor, No. 19&20, Street 106, Sangkat Wat Phnom, Khan Daun Penh, Phnom Penh 12100, Cambodia; alaillou@unicef.org (A.L.); epoirot@unicef.org (E.P.); 3 UMR-204 Nutripass, Institut de Recherche pour le Développement, IRD/UM/SupAgro, 34390 Montpellier, France; franck.wieringa@ird.fr (F.T.W.); jacques.berger@ird.fr (J.B.); 4 Department of Nutrition, Exercise and Sports, Faculty of Science, University of Copenhagen, 2200 Copenhagen, Denmark; nro@nexs.ku.dk

**Keywords:** feeding practices, nutritional status, child’s growth, wasting, stunting, Cambodia, longitudinal study

## Abstract

Age-appropriate feeding practice (ADF) during early childhood are vital for optimal nutrition. This longitudinal study determined the effect of selected risk factors and ADF, as described by the National Nutritional Recommendations, on linear and ponderal growth of children below 24 months of age. Weight and length measures were used to calculate z-scores of anthropometric measures by WHO standards. The prevalence of stunting increased from 13.2% to 32.4% over time, while prevalence of wasting remained stable (14.5%). At first visit, 43% of children of all ages complied with ADF criteria, a proportion which decreased to 7.1% in follow-up. The quality of feeding practices for children above 12 months of age was the poorest, where at the last visit, only 6% complied with the criteria for ADF. The linear mixed-effect models found the association between ADF and ponderal growth to be significant (weight-for-height estimate: 0.05 SD). In Cambodia, Ratanakiri province, ADF was the second largest determinant for ponderal growth. We recommend province specific public health actions. For children above 6 months, the quantity of food given needs to be increased, followed by the meal frequency. Mothers’ educational level, improved sanitation, and drinking water quality were among strongest predictors of a child’s growth.

## 1. Introduction

Malnutrition in the first five years of life, indicated by the presence of stunting or wasting, is associated with life-threatening consequences, as well as has a negative impact on physical and cognitive development [1,2,3,4]. These two forms of undernutrition—wasting and stunting—together with vitamin deficiencies and intrauterine growth restrictions were estimated in 2011 to account for 3.1 million deaths, with stunting alone was associated with 1.0 million deaths [2]. The latest Cambodian Demographic and Health Survey reported a national stunting prevalence of 32%, and 10% for wasting in children under five years of age, representing one of the highest rates in the region [5]. In certain provinces of Cambodia, stunting can affect up to 44% of children (Preah Vihear/Stung Treng province), while wasting prevalence reached the highest level of 15.1% in Otdar Meanchey province [5].

The growth of a child is influenced by several factors, of which adequate nutrient intake is among the most important. Age-appropriate feeding practices should satisfy both the energy and nutritional requirements of a healthy child and promote optimal growth and development. At minimum, a diet should at least prevent the occurrence of malnutrition [1,2,6,7,8]. Other factors such as indicators of household living standard or mothers’ educational level are perceived as underlying determinants of malnutrition and poor child growth. In Cambodia, poor infant and young child feeding practices together with low maternal education level, poverty, and a predominantly rural population were found to be main contributors to the poor nutritional status of children under five years of age [5]. Even though breastfeeding is a common feeding practice in Cambodia with 96% of children being ever breast-fed, the periods of exclusive and predominant breastfeeding are below recommendations, reaching an average of 3.7 months and 4.8 months respectively. The starting age and the poor quality of complementary feeding contribute to the inadequacy of feeding practices [5,9].

WHO Infant and Young Child Feeding (IYCF) indicators were developed to be able to assess trends and compare the quality of feeding practices globally. The indicators are separated into indicators of breastfeeding (exclusively below 6 months of age) and indicators of complementary feeding practices (6 to 24 months of age) [10,11]. IYCF indicators are used to mobilize public health actions by assessing the minimum diet of a child, ensuring access to a bare minimum of a variety of foods and of a number of meals per day [10,11]. These indicators have similar minimum diet requirements for all children between 6 and 24 months of age. However, during the first two years of life, the energy and nutritional requirements of a child increase with age, implying that caregivers need to adjust the feeding practices during this period. In studies with repeated data collections points, IYCF indicators are often avoided and researchers try to find alternatives [12].

The published evidence presents major difficulties in establishing a direct relation between IYCF indicators and the nutritional status of the child [13,14,15]. Out of all indicators, Minimum Diet Diversity (MDD) was the only one validated in an international sample and generally recognized as a reliable indicator for the quality of complementary feeding [11,16,17]. In comparison with other methods of assessment of feeding practices, IYCF indicators had a low sensitivity and specificity towards different forms of undernutrition, suggesting further improvements are needed [14,18,19]. While IYCF indicators are commonly used to assess the diet of children, very few studies have attempted to evaluate the appropriateness of the diet by age [20,21,22,23].

The overall aim of the present study was to estimate the effect of appropriate feeding practices on linear and ponderal growth of Cambodian children between the age of 0 and 2 years. Growth of the children was assessed through anthropometric measures and feeding practices through a constructed indicator for age-appropriate feeding practices based on breastfeeding and complementary feeding practices considering the recommendations published in the Cambodian National Nutrition Guidelines [24]. Secondly, the effect of relevant determinants, including the constructed indicator for age-appropriate feeding practices, on the children’s growth was evaluated.

## 2. Materials and Methods 

### 2.1. Study Design and Participants

Data were obtained from the longitudinal cohort study “MyHealth” project, under its original name “The Cambodian Health and Nutrition Monitoring Study”, which started its first data collection in March 2016. Ethical clearance was obtained from the Cambodian National Ethics Committee for Health Research of the Ministry of Health of Cambodia (Number 117/NECHR.).

The main objective of this prospective cohort study was to inform the government on achievements from enhanced health monitoring by analysing the gathered health and nutritional information, as well as changing trends over time. Six districts with afferent villages from three provinces in Cambodia were included—Russei Kaev in Phnom Penh, Chitr Borie and Krong Kratie in Kratie province, and Ou Chum, Krong Ban Lung, and Bar Kaev in Ratanakiri province. The provinces represent different population groups from Cambodia. The district in Phnom Penh concentrates a poor suburban population, Kratie is represented by a poor agriculture-dependent population, while the study population from Ratanakiri is representing ethnic groups practicing subsistence agriculture and food collection from wild sources.

The sample size at baseline was calculated to be of 1200 children under 2 years of age per province in order to detect a reduction in child stunting from 32% to 26% over a 3-year period, with a precision of 3% and a dropout of 20%. All the villages in selected districts were considered part of the study area. The village health support group, volunteers present in each village in Cambodia, supported our recruitment process by offering lists with all children under two years of age and pregnant mothers. All persons on these lists were asked to participate in the study. To test the persistency of the findings and to ensure an adequate sample size, pregnant women were invited to enroll their newborn children later in the study.

Informed formal consent was obtained from mothers or other legal caregivers of the participants, explaining in the local language the purpose of the study, potential risks and benefits, and the right to refuse or to withdraw from the study at any given point. Caregivers were present throughout data collection and responded to the survey questions. Furthermore, the confidentiality of information was assured for each participant including data protection and the use of personal identification numbers.

For this study, the first four rounds of follow-up were considered, counted from baseline to follow-up 3. Follow-ups were conducted almost every fourth month, leading to a maximum follow-up length of 1 year. The timeline is presented in the Supplementary Figure S1. Participants included later in the study were added to the study sample population. To level out the duration of participation, the timeline was described by the number of visits starting at recruitment.

**Figure 1:**
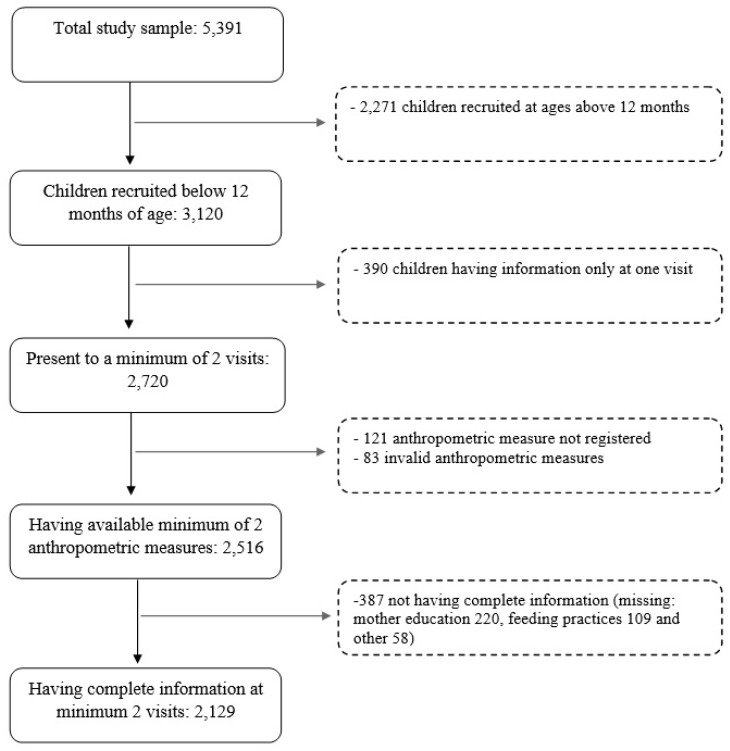
The population flow chart of selection of children for the study.

A child was included in the analysis when the following inclusion criteria were met: (1) being below 12 months of age at recruitment (*n* = 3120), (2) being present to a minimum of 2 follow-ups (*n* = 2720), (3) having available a minimum of two anthropometric measures (*n* = 2516), and (4) having complete information at minimum two visits (*n* = 2129). The figure below illustrates the selection process of the sample population (Figure 1). It should be noted that in this study, participants were allowed to be absent at visits.

### 2.2. Outcome Measures

Weight, length/height, and left mid-upper arm circumference (MUAC) were measured in duplicates for each child and the mean values were further used. Weights were recorded using calibrated digital scales (SECA 874, Hamburg, Germany) with 100 g precision. Recumbent lengths were measured to the nearest 1 mm using UNICEF boards with standing plates and moveable head boards. The UNICEF standard MUAC tape (S0145620 MUAC, Child 11.5 Red/PAC-50) was used with cut-off points from red to yellow at 115 mm and from yellow to green at 125 mm. Anthropometric variables were calculated according to the WHO Growth Child standards 2006 [25] as z-scores for length/height-for-age (HAZ; stunting), weight-for-age (WAZ; underweight), and weight-for-height/length (WHZ; wasting). Implausible values, as recommended by the WHO growth standards, (WHZ and WAZ below −5 or above +5 and HAZ below −6 or above +6) were converted to missing value [25]. Wasting, or acute malnutrition, was identified by WHZ at a level below −2 standard deviations (SD) and/or, for children above 6 months of age, MUAC measures below 125 mm. Stunting, indicating chronic malnutrition, was identified through HAZ at a level below −2 SDs. Categories identifying concurrent forms and optimal anthropometric measures were also considered during the study. Overweight and obesity, identified by a WHZ measure above +2 and +3 SDs, were not explored in this study as the prevalence was 0.6% for overweight and 0.1% for obesity.

An electronic tablet-based questionnaire was used to collect information on socio-economic status, diet including dietary diversity and breast-feeding practices, Water, Sanitation and Hygiene (WASH) practices, and other indicators. For more details regarding the questionnaires used, please see some earlier articles [26,27,28].

The Appropriate Daily Feeding (ADF) practices were classified according to the Cambodian National Nutrition Recommendation for infants’ and young children’s diet by age groups as presented in Table 1 [24]. The indicator was based on 24-recall information on breastfeeding practices and complementary feeding practices. Breastfeeding practices were assessed based on frequency and intake of other liquids or other semi-liquids foods. Complementary feeding practices were considered based on quantity of foods, measured in spoons or traditional bowls of 250 mL, and the frequency of meals. ADF was constructed to have a binary structure, whether the child adhered to the criteria for age-appropriate feeding practices or not. If information on feeding practices was not captured, missing values were computed. The minimum diet diversity (MDD) was evaluated based on the following seven food groups: (1) grains, roots, and tubers; (2) legumes and nuts; (3) vitamin A fruits and vegetables; (4) other fruits and vegetables; (5) meats; (6) eggs; and (7) dairy products [10,11,29]. Relevant to children aged more than 6 months, MDD was used for description purposes only in a binary format at a cut-off point of 4 out of 7 categories of food.

Other factors considered as determinants were maternal educational level, household size, household ownership of a latrine, source of drinking water, treatment of drinking water, province, age, gender of the child, and nutritional status. Age was calculated in months with two decimals. Gender was represented by categories of Male/Female. The maternal education level and a proxy of household size, the number of children in the household, were used as indicators for the living standard of the household. The maternal education level was stratified by categories: “No education”, “Primary education”, and “Secondary or above”. The number of children in the household was used as a proxy for the household size and is represented on a continuous scale from 1 to 10. The household ownership of a latrine had a binary form (“Yes” or “No”), where sharing with multiple households was coded as not owning a latrine [30]. The household consumption of safe drinking water was considered by the type of drinking water source, categorized as “Improved” and “Unimproved”, and whether the water was treated before consumption [30].

### 2.3. Statistical Analysis

The population from three provinces, Ratanakiri, Kratie, and Phnom Penh, was compared using information collected at the first visit. The categorical indicators are presented in proportion with the number of observations. The distribution between groups was tested with a Chi-square test for multiple proportions. Ordinal variables, such as age and number of children in the household, were represented through mean and standard deviation. The tests applied were non-parametrical methods—a Kruskal Wallis test with a post-hoc Wilcoxon test.

Follow-up data was analysed using generalized linear mixed-effect models for categorical variables and unadjusted linear mixed-effect models for ordinal ones. Using the visit number and child identification number as the dependent variable, the models could detect significant differences along the follow-up period.

Linear mixed-effects models were used to assess the effect of determinants on the linear and ponderal growth of the children. Selected indicators included, age, gender, province, age-appropriateness feeding practices, nutritional status, mother’s educational level, household size, ownership of latrine, and drinking water quality, were considered as risk factors for changes in children’s growth. The random effects were used to identify and match the data successively [31]. The random effects in this case were the personal identification number and the visit number, used as a proxy for the time between follow-ups. For this purpose, three models were constructed: an unadjusted model between outcome measures and ADF (A); a model adjusted for individual and socio-economic factors (B); and a fully-adjusted model including water and sanitation elements (C). The variance-covariance structure was unbalanced, while the best model was chosen based on the Akaike Information Criterion (AIC) and Bayesian Information Criterion (BIC). The fit of the models was compared using likelihood ratio tests. Changes in z-scores were assessed at the smallest unit change (0.01), based on the binary form of ADF for each visit. For the other variables, the reference groups were Ratanakiri as province, Female sex, not wasted nor stunted as nutritional status, no education for maternal education level, owning a latrine, and having unsafe drinking water for household. The continuous variables were compared at one-unit increase for age (+0.1 months), and number of children in the household (+1 child). Interaction between terms was tested when strong correlations were observed, as, for example, between province and ADF. Validation of the models was tested through the normality of residuals. F-tests were used to assess the significance of the estimates.

The z-scores were calculated from the anthropometric measures by using WHO Anthro software by using the growth standards for children between 0 and 60 months [32]. The estimations for linear mixed-effect models were done by maximum likelihood using R version 3.4.0, by engaging packages with statistical functions for longitudinal data (packages: Lmer4, lmerTest, nlme). Significance of specific estimates, as well as other tests, was considered for a *p*-value below 0.05 [33,34,35].

## 3. Results

The mean age of the children at recruitment, representing the first visit, was 6.19 months with a standard deviation (SD) of 3.30 months. The ratio of males to females was close to one (Table 2). Compared to the other provinces, the study population from Ratanakiri had a higher proportion of lower educated mothers (*p* < 0.001) and a bigger proportion of households without a latrine. For the quality of drinking water, households in Kratie used more often unimproved water sources, without treatment before drinking. Children from Ratanakiri had a higher prevalence of stunting (21.7%), while in Kratie wasting was more prevalent (20.4%) than in the other provinces. Children in Phnom Penh had a considerably better nutritional status, with 81.9% not being stunted or wasted.

The analysis of feeding practices revealed significant differences between provinces (Table 2). Predominantly breastfeeding of children under 6 months was a more common practice in Ratanakiri and Kratie than in the suburban district of Phnom Penh. Only 32.4% of children under 6 months from Phnom Penh received a minimum of six times per day breastfeeding without other liquid or semi-solid foods (*p* < 0.001). Adherence to ADF criteria was reached by 42.9% children of all ages, while 50.6% reached the criteria excluding quantity of complementary foods at first visit. The criteria of ADF was reached in the biggest proportion in Kratie, where almost 60% of children 6 to 9 months of age started with complementary feeding according with criteria described in Table 1. In addition, the breastfeeding practices for children below 6 months of age in Kratie reached the highest attainability for an age adequate diet, as compared to the other provinces. The quality of feeding practices in Phnom Penh were significantly lower than in the other two provinces, driven by both poor breastfeeding practices and by low frequency of meals.

Table 3 presents the information with complete cases collected during the visits. Slightly more than half of the children below 6 months of age (56% at first visit and 56.5% at second visit) were predominantly breastfed for a minimum of six times per day, as described by ADF. With a larger proportion of children moving towards weaning period, the proportion of children reaching criteria of ADF decreased considerably, as at the last visit, only 7.1% of children were having an age-appropriate diet. The quantity of complementary foods is one of the main factors that impacts the rate of achievement of age-appropriate diet. This could be especially observed at the fourth visit when the proportion of children adhering to all other criteria besides quantity was of 51.1%. After 9 months of age, children should receive at least a half of a bowl of food (1/2 of 250 mL) three times a day. In contrast, the diversity of diet improved with age, as MDD attainability increased from 20% to 35%.

In terms of nutritional status, the prevalence of stunting increased from 13.7% to 30.7% as children got older. The prevalence of wasting remained stable, affecting between 13% and 15% of children. Slightly more than half of the population (54.4%) were never identified as stunted or wasted, while approximately 70% of children remained or became stunted between data collection visits.

**Figure 2:**
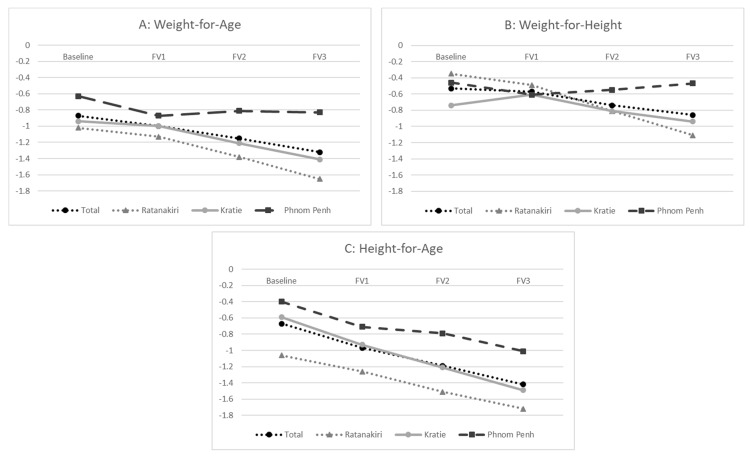
Means for z-score of anthropometric measures: Weight-for-Age (**A**), Weight-for-Length (**B**) and Length-for-Age (**C**) for the total population, Ratanakiri, Kratie, and Phnom Penh.

Changes in z-scores of anthropometric changes over time are illustrated in Figure 2. HAZ showed the greatest change over time with an average of −0.75 SD, followed by WAZ with −0.45 SD and WHZ with −0.33 SD. While there were differences in HAZ scores and stunting prevalence among the provinces, the change of time in HAZ scores was similar. In contrast, WHZ scores remained stable in Phnom Penh, whereas they decreased in Kratie and Ratanakiri.

The investigation of effects of feeding practices on growth using linear mixed-effect models showed a significant effect between ADF, the created indicator for feeding practices, and the z-score ratio between weight and height (estimate: 0.06, Standard Error = 0.02, *p* = 0.02). This suggests that feeding practices play an important role on the balance between weight and height gain, called ponderal growth, during the first 2 years of life. A summary of the results can be seen in Table 4 and fully presented in the Supplementary Tables. The strongest predictors for undernutrition were low maternal education level and lack of ownership of a latrine. Mothers enrolled in secondary school or a higher education level had positive association with growth indicators (WHZ estimate: +0.22 SDs, SE = 0.06, *p* < 0.001; HAZ estimate: +0.34 SD, SE = 0.07, *p* < 0.001). The ownership of a latrine was also associated with improved growth, with not owning a latrine being associated with a negative effect on HAZ (estimate: −0.15 SDs, SE = 0.04, *p* < 0.001) and WHZ (estimate: −0.10 SDs, SE = 0.04, *p =* 0.01).

Since provinces showed significant contrasts in many aspects of study population characteristics and since had an interaction with ADF, fully adjusted linear mixed-effect models were ran for each province (Table 5). In these models, maternal educational level and latrine remained important predictors for the growth of children in the provinces of Kratie and Ratanakiri. The ADF was the second strongest predictor for improved WHZ measures (estimate: +0.13 SDs, SE =0.04, *p* < 0.001) after maternal education level. In Kratie, sanitation was one of the strongest predictors. The results of models for the province of Phnom Penh did not significant associations between indicators of growth and the selected risk factors, suggesting that in suburban areas, other factors might be determinants of growth.

## 4. Discussion

This study highlights an increasing prevalence of stunting with age, especially in Ratanakiri province situated in the North Eastern region of Cambodia. To estimate the impact of appropriate feeding of the child over an age-span from exclusive breastfeeding through the complementary feeding period to a family diet, on the occurrence of malnutrition, we created the indicator for ADF. To the best of our knowledge, this is the first study considering feeding practices from the perspective of appropriateness for age including the quantity of complementary meals as part of the indicator’s criteria. For children above 6 months, not only the quality of complementary feeding needs to be improved, but also the quantity of food given during meals and the frequency of meals. Furthermore, factors related to household living standard and to hygiene, such as mothers’ educational level and improved sanitation and drinking water quality are among the strongest predictors of child’s growth rate. For younger children, enhanced breastfeeding practices are needed, especially for the suburban area of Phnom Penh. The breastfeeding practice reached the criteria of age-appropriate feeding practice in a large proportion where 56% of children between 0 and 6 months of age were predominantly breastfed with a minimum of six breastfeeding sessions per day. However, we have to raise awareness about the poor breastfeeding practices among the population from suburban area of Phnom Penh, where only one third reached the criteria for appropriate breastfeeding practice for children under 6 months of age. The increase in the use of commercial baby foods and infant formula might be one of the drivers for the poor achievement of breastfeeding practices [36].

During the weaning period, the transition from breastfeeding to a normal diet and the adherence to the criteria for appropriate daily feeding practices decreases significantly. At the first visit, 30.5% of children between 6 and 12 months old had age-appropriate feeding practices, which decreased to 7.2% at the last visit. Even though most of them are partly breastfed after 9 months of age, the frequency of meals and quantity during complementary feeding was considered inadequate for the age, according to the National Nutrition Recommendations. The adherence to age-appropriate feeding practices was especially impacted by the criteria of quantity of complementary foods, which emphasize on the fact that the children received too little food for their age. A study from Ethiopia attempted to assess the diet appropriateness of the children by using the classic IYCF indicators including the indicators for: age of introducing complementary foods, a minimum diet diversity and minimum meal frequency [37]. The authors report a minimum meal frequency being achieved by 67.3% for the children, while the addition of MDD to the overall score decreased the percentage to only 9.5%. Nevertheless, they found that older children, above 12 months of age, were three times more likely to have a minimum diet diversity when compared to the age group of 6 to 12 months of age. This study emphasized the fact that even though the older children were most likely to reach the criteria for a minimum diet diversity, that does not imply that the children reached the real bare minimum energy and nutrient intakes as the proportion of undernourished children increased for older age groups. Hence, assessing the diet of infants and young children by criteria increasing with age allows for a better evaluation of the diet children are receiving.

We found significant associations between age-appropriate feeding practices and ponderal growth expressed though positive changes in Weight-for-Height/Length (WHZ). For the follow-up period of 1 year, it can be estimated that an age-appropriate feeding practices, as described in Table 1, would add +0.2 SDs to the overall anthropometric ratio between weight and height. This would prevent the development of acute malnutrition and would promote an optimal growth. Available studies generally measure the feeding practices for infants and young children by Minimum Acceptable Diet (MAD), the main IYCF index combining Minimum Meal Frequency and Minimum Diet Diversity indicators. As compared to our study, MAD is in general found to be associated with measures of Length/Height-for-Age, an indicator used to identify cases stunting (HAZ) [14,16], an association identified to be the result of including MDD in the index, as suggested by studies [14]. MDD itself was found to have an association with HAZ and to be an indicator for the quality of diet [16,38,39]. Through the strong relation with ponderal growth, the created indicator for feeding practices, ADF, seems to be more sensitive to total energy intake than nutrient intake. In the short-term, the effect of energy intake has a more direct influence on ponderal growth measures than linear growth (HAZ). Therefore, we suggest future studies to consider the feeding practices from criteria that shall be increased adequately with child’s nutrient requirements by age.

The disaggregation of the final models by province identifies determinants to be specific for each province. In Phnom Penh, we would recommend future public health actions to promote breastfeeding practice, including the promotion of an appropriate number of breastfeeding sessions. This could be especially relevant for lower educated mothers. In both Kratie and Ratanakiri, our study suggests that improvements in the quantity of complementary foods would improve the overall adherence to ADF. This should be especially targeted in Ratanakiri, where the feeding practices are the second most important predictor for ponderal growth, an indicator of wasting.

There are a few factors that can be considered for explaining the province specific findings. The first explanation would be that feeding practices for infants and young children in the three regions are different. In Phnom Penh, where the population finds it easier to purchase infant formula and the mothers return to factory work soon after delivery, children might not receive the best breastfeeding practice and it might be replaced by substitute milk or semi-solid foods. Children living in Kratie province seemed to have a better diet adequate for their age, even though this could be further improved through the promotion of breastfeeding practices. Here, the complementary feeding practices seemed to be improved as compared with the population from the other two provinces, possibly for reasons related food security.

Another explanation for the difference among provinces is the fact that different factors contribute in different ways to a faltering growth. While feeding practices play a significant role for the population for Ratanakiri, the other two provinces are seeing an increase in other factors such as quality of drinking water and sanitation; these two factors might increase the risk of infections and contribute negatively to child’s growth. A very important predictor—mother’s educational level—has a different strength of association between the three provinces. The strength was the highest in Ratanakiri, suggesting that literacy of the mothers as well as the household living standards are the main drivers of child’s growth rate. A study from Zambia [16] found MDD and linear growth to be associated, while maternal education level had the role of mediating the negative impact of poor diet diversity. This means that for highly educated mothers, the growth of the child is not influenced in the same degree as for uneducated mothers by the feeding practices. Mothers with higher educational level might have access to other information, services and materials to promote an optimal child’s growth, while the household living standard might be improved. The evidence presented above, including this study, supports the fact that efficient intervention to improve the nutritional status of children should not rely solely on activities targeting improvements of feeding practices, but should rather adopt a holistic view on health and nutrition [16,40,41].

The adjustments for nutritional status assessing stunting and wasting shows findings that suggests a possible relation between forms of undernutrition. Children suffering from acute malnutrition encounter a slower increase in height, which could lead to occurrence of stunting. Estimates of the impact of wasting on HAZ and of stunting on WHZ values suggest a possible interaction between forms of malnutrition, where the presence of either form of undernutrition would increase the risk of developing concurrent forms of undernutrition. The relation between acute (wasting) and chronic (stunting) malnutrition was explored by other papers where undernourished children faced difficulties in obtaining a long-term optimal nutritional status [38,42,43]. Combined, these findings emphasize the need to build an integrated approach towards undernutrition, distinguishing by degree of complexity or severity rather type [44,45,46]. Nevertheless, further research is needed to understand how this can be best implemented in low- and middle-income countries.

The core strength of this study relates to the cohort design that includes repeated measures of a large number of participants located in three distinctive provinces in Cambodia, that can help to describes population trends and to produce valuable evidence. The methods used for data collection, including the anthropometric assessments, ensured the accuracy of measurements at international standards. The potential to improve the IYCF indicators was perceived from the possibility of incorporating multiple perspectives of breast-feeding (frequency) and complementary feeding (frequency and quantity), while maintaining a similar methodology. The possibility of validation between answers ensured the accuracy of variables.

We acknowledge the limitations of this study. As is known, the population from the South East region of Asia has lower standards for growth than the rest of the world, but with no other standard available for the region, WHO growth standards were used. One of the most common issues among repeated measures is the unknown changes occurring between data collections, adding to the limitations of 24-h recall period of the feeding practices. This implies that the entire dimension of feeding practices, as well as the presence of other risk factors, cannot be fully captured. Additionally, the risk of misclassification and social desirability biases should be also considered. Lastly, it should be once again mentioned that the estimates are the results of fitted models, hence having an estimate available for entire population, as well as the fact that it cannot be ensured that missing values are not moderating the value of estimates [47].

## 5. Conclusions

In Cambodia, the prevalence of undernutrition for children under five remains a public health concern for society. This study revealed low adherence of child feeding practices to the Cambodian National Nutrition Guidelines, becoming a concerning issue for children above 12 months of age. Ponderal growth, as showed by Weight-for-Height, was significantly influenced by age-appropriate feeding practices, which could indicate an appropriate energy intake for the age of the child.

Indicators of household living standards including mother’s education level and access to good hygienic conditions are likely to present proxies to a better food security and possess less risk of experiencing infectious diseases. Hence, in the analysis, these indicators are being stronger drivers of undernutrition in Cambodia as compared to feeding practices. Therefore, we would suggest a number of public health activities starting with campaigns targeting increased awareness among communities to better identify faltering growth, followed by activities to improve the educational level among women and improve sanitation across the country. Lastly, the quality of complementary feeding practices should be enhanced from the meal frequency and quantity of foods, while in Phnom Penh there is a need for improvements of breastfeeding practices.

## Figures and Tables

**Table 1:** The construct of indicator for age-appropriate feeding practices (ADF) based on breast-feeding and complementary feeding practices.

Age Group	Breast-Feeding	Complementary Feeding	
		Frequency	Quantity ^1^
0–5.9 months	Predominant breast-feeding at least 6 times per day	No complementary feeding	
6–8.9 months	Breast-feeding at least 6 times per day (complementary)	Maximum 3 meals per day	2–3 tablespoons to half Chan Chang Koeh for each meal
9–11.9 months	Breast-feeding at least 6 times per day (complementary)	Minimum of 3 meals per day	From half to a full Chan Chang Koeh for each meal
12 months+	Breast-feeding at least 3 times per day(complementary)	Minimum of 3 meals per day	Minimum half of a full Chan Chang Koeh for each meal

^1^ Chan Chang Koeh, a traditional South Asian bowl equivalent to 250 milliliters; one tablespoon is equivalent to 10 milliliters [24].

**Table 2:** Descriptive characteristics of study population at the first round of visits.

Variable	Total *n* = 2129	Ratanakiri *n* = 682	Kratie *n* = 799	Phnom Penh *n* = 648	*p*-Value
Age at recruitment	6.19 (3.30)	5.87 (3.33)	6.22 (3.22)	6.48 (3.33)	0.005
Age group					
0–5.9 months	48.4% (1030)	53.1% (362)	47.4% (379)	44.6% (289)	
6–8.9 months	27.2% (580)	24.4% (167)	29.6% (236)	27.3% (177)	
9–11.9 months	24.3% (519)	22.4% (153)	23.0% (184)	28.1% (182)	
Sex: Male	50.5% (1076)	47.9% (327)	50.6% (405)	53.1% (344)	0.08
Breast-feeding status ^1^					<0.001
Predominantly	34.3% (731)	43.2% (287)	38.9% (303)	24.2% (141)	
Partly	51.9% (1105)	51.5% (342)	57.5% (448)	54.0% (315)	
None	8.9% (190)	5.3% (35)	3.6% (28)	21.8% (127)	
NA’s	(103)	(18)	(20)	(65)	
ADF	43.0% (845)	47.7% (306)	52.5% (399)	25.0% (148)	<0.001
0–5.9 months	55.9% (538)	62.8% (221)	65.4% (237)	32.4% (80)	
6–8.9 months	45.6% (240)	29.5% (60)	59.2% (132)	27.1% (48)	
9–11.9 months	14.1% (67)	18.2% (25)	16.7% (29)	7.9% (13)	
NA’s	(166)	(40)	(40)	(85)	
Minimum Diet Diversity ^2^	19.1% (204)	21.7% (68)	17% (75)	21.8% (74)	0.03
Nutritional status					<0.001
Not stunted nor wasted	74.3% (1573)	70.4% (477)	71.5% (565)	81.9% (531)	
Stunted	13.8% (295)	21.7% (147)	11.6% (92)	9.0% (56)	
Wasted	14.8% (315)	12.4% (84)	20.4% (161)	10.6% (69)	
NA’s	(14)	(5)	(9)	(0)	
Mother education					<0.001
No education	23.0% (490)	42.8% (292)	16.8% (134)	10.0% (65)	
Primary	40.2% (855)	32.3% (225)	48.7% (389)	37.4% (241)	
Secondary and more	39.6% (780)	24.2% (165)	34.5% (276)	52.6% (339)	
NA’s	(3)	(0)	(0)	(3)	
Number of children	2.4 (1.37)	2.59 (1.39)	2.37(1.28)	2.30 (1.31)	0.08
NA’s	(13)	(1)	(6)	(6)	
Safe drinking water	93.4% (1931)	97.5% (659)	84.4% (666)	99.3% (4)	0.04
NA’s	(63)	(10)	(12)	(41)	
Do not have latrine	40.0% (836)	52.9% (361)	57.9% (463)	2.0% (13)	<0.001
NA’s	(40)	(0)	(0)	(40)	

^1^ Predominant—Exclusive breast-feeding including water, Partly—Breast-feeding and Complementary feeding. ^2^ MDD valid for children above 6 months of age. NA’s—Missing values. ADF – Age-approriate feeding practices.

**Table 3:** Changes in feeding practices and prevalence of undernutrition along the study timeline for cases with complete information.

Variable (%)	1st Visit *n* = 1898	2nd Visit *n* = 1332	3rd Visit *n* = 1212	4th Visit *n* = 1091	*p*-Value
Age group					<0.001
0–5.9 months	49.0% (931)	22.4% (299)	0% (0)	0% (0)	
6–8.9 months	26.7% (506)	26.7% (355)	12.9% (157)	0% (0)	
9–11.9 months	24.3% (461)	28.6% (381)	27.3% (331)	6.1% (67)	
12 months+	0% (0)	22.3% (297)	59.7% (724)	93.9%(1024)	
Province					0.18
Ratanakiri	33.0% (627)	36.0% (480)	34.9% (423)	34.4% (276)	
Kratie	38.5% (732)	40.4% (538)	38.5% (467)	41.5% (453)	
Phnom Penh	28.4% (539)	23.6% (314)	26.6% (322)	24.0% (262)	
Breast-feeding status ^1^					<0.001
Predominantly	37.1% (705)	16.7% (223)	1.2% (15)	1.6% (18)	
Partly	53% (1006)	77.9% (1037)	88.2% (1059)	72.9% (796)	
None	9.9% (187)	5.4% (72)	10.6% (128)	25.4% (277)	
ADF excluding quantity ^2^	50.6% (960)	61.3% (816)	61.5% (745)	51.1% (558)	<0.001
ADF	42.9% (815)	35.9% (479)	17.7% (214)	7.1% (70)	<0.001
0–5.9 months	55.9% (520)	56.5% (169)	0% (0)	0% (0)	
6–8.9 months	45.7% (231)	48.2% (171)	56% (88)	0% (0)	
9–11.9 months	13.9% (64)	25.7% (98)	17.2% (57)	14.9% (10)	
12 months+	0% (0)	13.8% (41)	9.5% (69)	5.9% (60)	
Minimum Diet Diversity ^3^	20.0% (194)	26.9% (278)	37.1% (449)	34.9% (424)	<0.001
Nutritional status					<0.001
Not stunted nor wasted	74.9% (1423)	71.8% (957)	68.2% (826)	61.6% (672)	
Stunting	13.2% (250)	18.9% (252)	24.5% (297)	32.4% (354)	
Wasting	13.6% (259)	14.7% (196)	13.7% (166)	15.4% (168)	
Mother education					0.04
No education	23.2% (440)	23.2% (309)	25.3% (307)	26.2% (286)	
Primary	39.8% (756)	40.2% (550)	40.4% (490)	40.1% (437)	
Secondary and more	36.9% (702)	36.6% (473)	34.2% (415)	33.7% (368)	
Safe drinking water	93.6% (1777)	92.9% (1235)	85.2% (1033)	78.8% (860)	<0.001
Do not have latrine	39.9% (759)	41.2% (559)	41.2% (499)	41.8% (456)	0.06

^1^ Predominant—Exclusive breast-feeding including water, Partly—Breast-feeding, and Complementary feeding. ^2^ Age-appropriate feeding practices (ADF) criteria. ^3^ MDD valid for children above 6 months of age.

**Table 4:** The effect of age-appropriate daily feeding practices on the z-scores of anthropometric measures for Weight-for-Age (WAZ), Weight-for-Height (WHZ) and Height-for-Age (HAZ), is illustrated by estimates of the linear mixed effect models, together with Standard Error (S.E.) and *p*-value (*P*.).

	Model A			Model B			Model C		
	*Est.*	*S.E.*	*P.*	*Est.*	*S.E.*	*P.*	*Est.*	*S.E.*	*P.*
WAZ	0.11	0.02	<0.001	0.004	0.01	0.8	0.004	0.02	0.83
HAZ	0.17	0.02	<0.001	0.01	0.02	0.91	0.01	0.02	0.90
WHZ	0.15	0.02	<0.001	0.06	0.02	0.02	0.06	0.02	0.02

Model A—unadjusted model; Model B—adjusted for individual and socio-economic indicators; Model C—fully adjusted.

**Table 5:** The effect of appropriate daily feeding practices (ADF) in fully-adjusted models separated by province. Table shows the linear mixed effect model estimates, together with Standard Error (S.E.) and *p*-value (*P*.).

Fully-Adjusted Model(C)	Ratanakiri			Kratie			Phnom Penh		
	*Est.*	*S.E.*	*P.*	*Est.*	*S.E.*	*P.*	*Est.*	*S.E.*	*P.*
WAZ	0.05	0.03	0.12	0.001	0.03	0.88	0.02	0.04	0.19
HAZ	0.01	0.04	0.79	0.04	0.03	0.19	−0.08	0.05	0.07
WHZ	0.13	0.04	<0.001	0.01	0.04	0.81	0.02	0.05	0.72

## Supplementary Materials

The following are available online at https://www.mdpi.com/2072-6643/12/1/12/s1, Figure S1: Timeline of MyHealth between Baseline and Follow-up 3, Table S1: Parameter estimates (Est.), Standard error (SE) and p-values of linear mixed effects models: Weight-for-Age by ADF; Table S2: Parameter estimates (Est.), Standard error (SE) and p-values of linear mixed effects models: Height-for-Age by ADF; Table S3: Parameter estimates (Est.), Standard error (SE) and p-values of linear mixed effects models: Weight-for-Height by ADF.
