# Supplementary material for: Age-Appropriate Feeding Practices in Cambodia and the Possible Influence on the Growth of the Children: A Longitudinal Study
Source: Nutrients. 2019 Dec 19;12(1):12. doi: 10.3390/nu12010012 (PMC7019767; doi:10.3390/nu12010012)
Supplement: Supplementary file 1 [file nutrients-12-00012-s001.pdf]

Supplementary material

Supplementary figure S1. Timeline of MyHealth between Baseline and Follow-up 3.

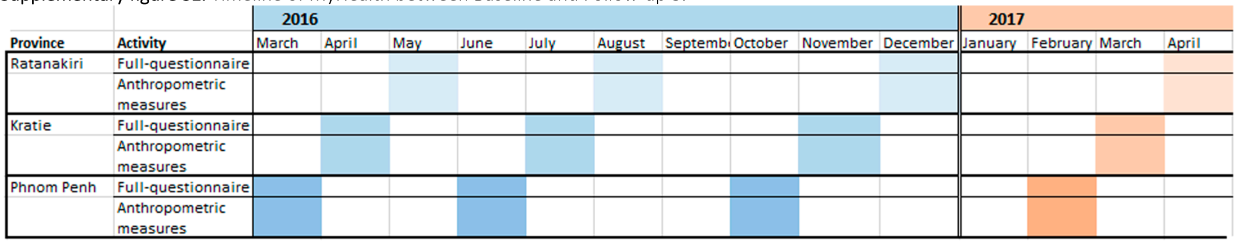

Supplementary table S1. Parameter estimates (Est.), Standard error (SE) and p-values of linear mixed effects models for Weight-for-Age by risk factors, including age-appropriate feeding practices

	Model A			Model B			Model C			Ratanakiri			Kratie			Phnom Penh		
**Variables**	**Est**	**SE**	***P***	**Est**	**SE**	***P***	**Est**	**SE**	***P***	**Est**	**SE**	***P***	**Est**	**SE**	***P***	**Est**	**SE**	***P***
**Intercept**	-1.04	0.02	< .001	-0.79	0.05	< .001	-0.74	0.06	< .001	-0.84	0.10	< .001	-0.69	0.10	< .001	-0.33	0.41	0.42
**ADF**	0.11	0.02	< .001	0.004	0.01	0.8	0.004	0.02	0.83	0.05	0.03	0.12	0.01	0.03	0.88	0.02	0.04	0.19
**Sex: Male**				-0.07	0.04	0.04	-0.08	0.04	0.03	-0.11	0.06	0.08	-0.03	0.06	0.56	-0.09	0.07	0.16
**Age**				-0.03	0.01	< .001	-0.03	0.01	< .001	-0.03	0.001	< .001	-0.03	0.003	< .001	-0.02	0.001	< .001
**Wasting**				-0.68	0.02	< .001	-0.69	0.02	< .001	-0.63	0.04	< .001	-0.69	0.04	< .001	-0.76	0.06	< .001
**Stunting**				-0.54	0.02	< .001	-0.56	0.03	< .001	-0.53	0.04	< .001	-0.42	0.04	< .001	-0.83	0.06	< .001
**Mother education: Primary**				0.18	0.05	< .001	0.15	0.05	< .001	0.24	0.07	< .001	0.13	0.08	0.11	-0.07	0.11	0.50
**Mother education: Secondary +**				0.32	0.05	< .001	0.28	0.05	< .001	0.51	0.08	< .001	0.23	0.09	0.01	0.02	0.11	0.83
**Household size**				-0.02	0.01	0.02	-0.02	0.01	0.04	-0.01	0.01	0.46	-0.02	0.01	0.19	-0.02	0.02	0.28
**Not having latrine**							-0.11	0.03	< .001	-0.09	0.05	0.06	-0.13	0.04	< .001	-0.13	0.17	0.43
**Safe drinking water**							0.03	0.03	0.26	0.001	0.04	0.94	0.05	0.03	0.16	0.11	0.39	0.78
**Province: Kratie**				0.01	0.04	0.88	0.03	0.04	0.56									
**Province: Phnom Penh**				0.30	0.05	< .001	0.26	0.05	< .001									
**Number obs/participants**	5,533/2,129									1,906/682			2,190/799			1,437/648		
**AIC/BIC values**	12,254/12,293			11,142/11,241			10,681/10,794			3,476/3,558			4,116/4,202			3,056/3,135		

ADF- Age-appropriate feeding practices; Reference groups: Not reaching criteria for ADF, Female, Not wasted, Not Stunted, No mother education, Owning a latrine, Not having safe water for household consumption, Ratanakiri province

Supplementary table S2. Parameter estimates (Est.), Standard error (SE) and p-values of linear mixed effects models for Height-for-Age by risk factors, including age-appropriate feeding practices

	Model A			Model B			Model C			Ratanakiri			Kratie			Phnom Penh		
**Variables**	**Est**	**SE**	***P***	**Est**	**SE**	***P***	**Est**	**SE**	***P***	**Est**	**SE**	***P***	**Est**	**SE**	***P***	**Est**	**SE**	***P***
**Intercept**	-1.14	0.03	< .001	-0.76	0.07	< .001	-0.69	0.09	< .001	-0.92	0.12	< .001	-0.45	0.14	< .001	0.24	0.54	0.66
**ADF**	0.17	0.02	< .001	0.01	0.02	0.91	0.01	0.02	0.90	0.01	0.04	0.79	0.04	0.03	0.19	0.05	0.05	0.07
**Sex: Male**				-0.09	0.05	0.06	-0.09	0.05	0.06	-0.19	0.08	0.02	-0.05	0.08	0.56	-0.05	0.09	0.59
**Age**				-0.06	0.002	< .001	-0.06	0.002	< .001	-0.05	0.002	< .001	-0.07	0.002	< .001	-0.07	0.01	< .001
**Wasting**				-0.07	0.03	0.07	-0.07	0.03	0.07	-0.05	0.05	0.29	-0.08	0.05	0.07	-0.09	0.08	0.10
**Mother education: Primary**				0.19	0.06	< .001	0.15	0.07	0.02	0.20	0.10	0.04	0.17	0.11	0.13	0.07	0.15	0.18
**Mother education: Secondary +**				0.40	0.07	< .001	0.34	0.07	< .001	0.65	0.11	< .001	0.39	0.12	< .001	0.08	0.15	0.38
**Household size**				-0.01	0.01	0.48	-0.01	0.01	0.48	0.001	0.01	0.80	0.01	0.02	0.43	-0.02	0.02	0.31
**Not having latrine**							-0.15	0.04	< .001	-0.16	0.06	0.01	-0.12	0.05	0.02	-0.19	0.23	0.41
**Safe drinking water**							0.04	0.03	0.24	0.08	0.05	0.13	0.01	0.04	0.79	0.06	0.52	0.91
**Province: Kratie**				0.22	0.06	< .001	0.24	0.06	< .001									
**Province: Phnom Penh**				0.56	0.07	< .001	0.50	0.07	< .001									
**Number obs/participants**	5,533/2,129									1,906/682			2,190/799			1,437/648		
**AIC/BIC values**	14,690/14,729			13,900/13,992			13,888/13,994			4,579/4,657			5,242/5,322			4,007/4,081		

ADF- Age-appropriate feeding practices; Reference groups: Not reaching criteria for ADF, Female, Not wasted, Not Stunted, No mother education, Owning a latrine, Not having safe water for household consumption, Ratanakiri province

Supplementary table S3. Parameter estimates (Est.), Standard error (SE) and p-values of linear mixed effects models for Weight-for-Height by risk factors, including age-appropriate feeding practices

	Model A			Model B			Model C			Ratanakiri			Kratie			Phnom Penh		
**Variables**	**Est**	**SE**	***P***	**Est**	**SE**	***P***	**Est**	**SE**	***P***	**Est**	**SE**	***P***	**Est**	**SE**	***P***	**Est**	**SE**	***P***
**Intercept**	-0.78	0.02	< .001	-0.48	0.07	< .001	-0.46	0.08	< .001	-0.38	0.11	< .001	-0.60	0.13	< .001	-0.43	0.50	0.39
**ADF**	0.15	0.02	< .001	0.06	0.02	0.02	0.06	0.02	0.02	0.13	0.04	< .001	0.01	0.04	0.81	0.02	0.05	0.72
**Sex: Male**				-0.09	0.04	0.04	-0.09	0.04	0.04	-0.02	0.07	0.82	-0.04	0.07	0.56	-0.19	0.08	0.01
**Age**				-0.03	0.001	< .001	-0.03	0.001	< .001	-0.06	0.001	< .001	-0.02	0.001	< .001	0.001	0.001	0.30
**Wasting**				-0.07	0.03	0.07	-0.07	0.03	0.07	-0.07	0.03	0.07	-0.06	0.04	0.09	-0.07	0.07	0.32
**Mother education: Primary**				0.16	0.05	< .001	0.14	0.06	0.01	0.26	0.08	< .001	0.13	0.09	0.18	-0.04	0.13	0.76
**Mother education: Secondary +**				0.25	0.06	< .001	0.22	0.06	< .001	0.38	0.09	< .001	0.17	0.10	0.09	0.06	0.13	0.66
**Household size**				-0.02	0.01	0.08	-0.02	0.01	0.08	0.01	0.02	0.58	-0.05	0.02	< .001	-0.03	0.02	0.09
**Not having latrine**							-0.10	0.04	0.01	-0.09	0.06	0.10	-0.11	0.05	0.04	-0.13	0.20	0.24
**Safe drinking water**							0.06	0.04	0.13	0.01	0.06	0.81	0.05	0.05	0.32	0.08	0.48	0.87
**Province: Kratie**				-0.17	0.05	< .001	-0.15	0.05	< .001									
**Province: Phnom Penh**				0.09	0.06	0.09	0.05	0.06	0.37									
**Number obs/participants**	5,533/2,129									1,906/682			2,190/799			1,437/648		
**AIC/BIC values**	14,167/14,207			14,004/14,097			13,900/13,909			4,665/4,737			5,618/5,698			3,635/2,708		

ADF- Age-appropriate feeding practices; Reference groups: Not reaching criteria for ADF, Female, Not wasted, Not Stunted, No mother education, Owning a latrine, Not having safe water for household consumption, Ratanakiri province
